# Sex-specific associations between carotid plaque and bone mineral density in patients with type 2 diabetes: a retrospective cross-sectional study

**DOI:** 10.1186/s13293-026-00830-y

**Published:** 2026-01-28

**Authors:** Bing Liu, Jun Chen, JingBo He, Qing Xue, Yue Liu, Fei Gao, Hao Qi

**Affiliations:** 1 Department of Endocrinology, First Hospital of Shanxi Medical University, No. 85 Jiefang South Road, Taiyuan, Shanxi Province 030001 People’s Republic Of China; 2 Department of Nuclear Medicine, First Hospital of Shanxi Medical University, Taiyuan, Shanxi Province China; 3 Department of Endocrinology, Shanghai Pudong New Area People’s Hospital, 490 chuanhuan South Road, Shanghai, 201299 China; 4 The Public Health College of Shanxi Medical University, Taiyuan, Shanxi Province China; 5 The First Clinical Medical College of Shanxi Medical University, Taiyuan, Shanxi Province China

**Keywords:** Bone mineral density, Carotid plaque, Type 2 diabetes mellitus, Carotid ultrasonography, Osteoporosis

## Abstract

**Background:**

Carotid plaque and osteoporosis commonly coexist in type 2 diabetes mellitus (T2DM), but whether their association differs by sex remains unclear. We examined sex-specific differences in bone measures and the adjusted association between carotid plaque and osteoporosis status in patients with T2DM.

**Methods:**

This retrospective cross-sectional study included 1,224 patients with T2DM (794 women and 430 men). Carotid plaque was assessed by ultrasound. Lumbar spine bone mineral density (BMD) and T-scores were measured by dual-energy X-ray absorptiometry (DXA), and osteoporosis status was categorized as normal bone mass, osteopenia, or osteoporosis. Clinical characteristics, bone measures, and osteoporosis prevalence were compared between patients with and without carotid plaque within each sex. Sex-stratified ordinal logistic regression models were used to evaluate the adjusted association between carotid plaque and osteoporosis categories with stepwise adjustment for BMI, diabetes duration, lipid parameters, and glycemic indices.

**Results:**

In men, patients with carotid plaques showed a modestly higher lumbar spine BMD (*P* = 0.013); however, lumbar spine T-scores and osteoporosis prevalence were similar between plaque and non-plaque groups, and no adjusted association with osteoporosis category was observed across models. In women, lumbar spine BMD, T-scores, and osteoporosis prevalence were similar between plaque and non-plaque groups in unadjusted comparisons. However, in women, carotid plaques were independently associated with higher odds of worse osteoporosis category after adjustment (Model 1: odds ratio (OR) 1.40 [95% confidence interval (CI) 1.05–1.85]; Model 2: OR 1.45 [95% CI 1.09–1.92]; Model 3: OR 1.50 [95% CI 1.12–1.99]; all *P* < 0.05).

**Conclusion:**

Among patients with T2DM, carotid plaque was independently associated with worse osteoporosis category in women but not in men. These findings indicate sex-specific associations between carotid atherosclerosis and skeletal health; however, given the cross-sectional design and unavailable key confounders (e.g., lifestyle factors, central adiposity measures, medication patterns, and fracture outcomes), the results should be interpreted as associative and require confirmation in prospective studies.

**Graphical abstract:**

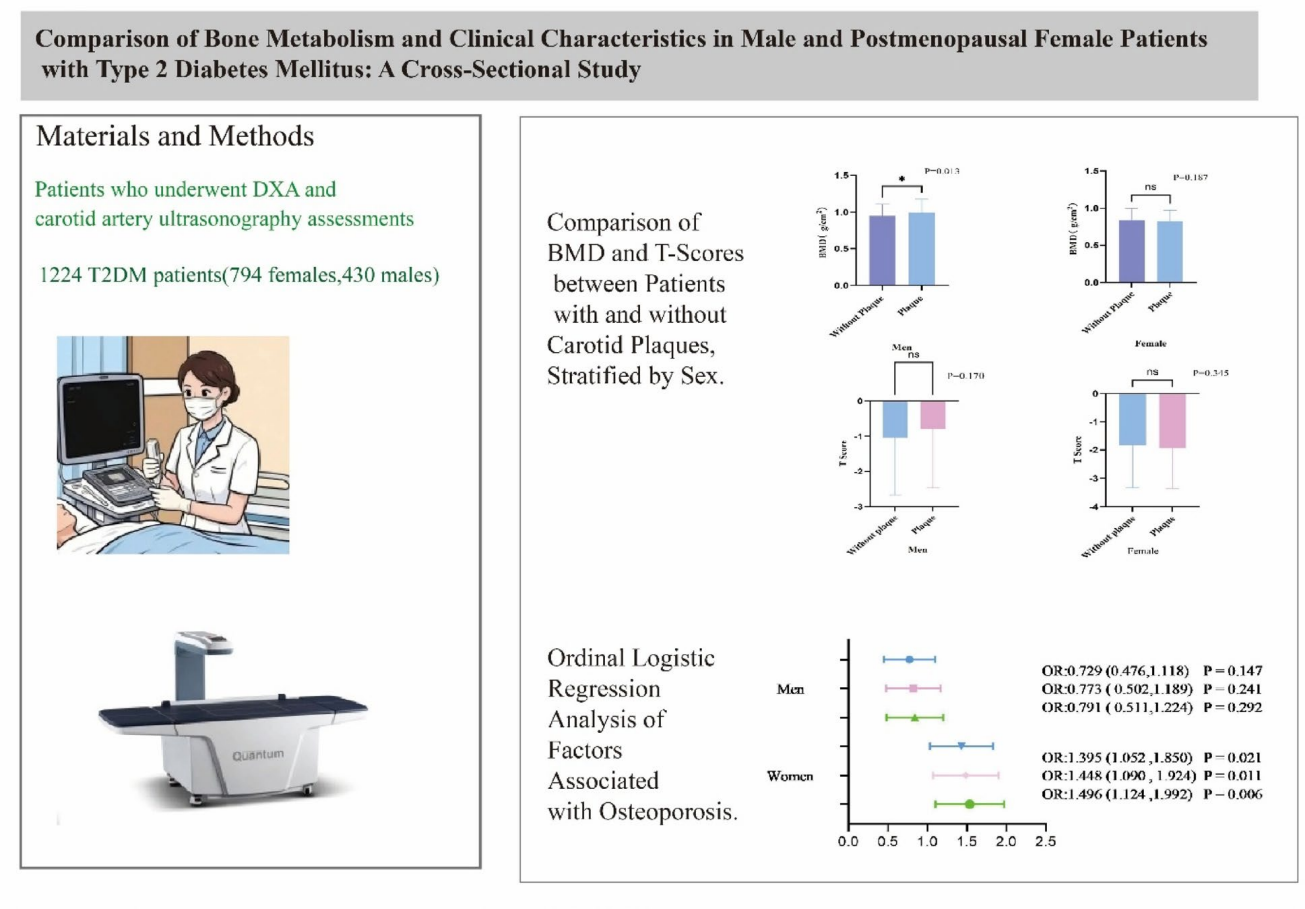

**Supplementary Information:**

The online version contains supplementary material available at 10.1186/s13293-026-00830-y.

## Abstract (plain language summary)

People with type 2 diabetes often develop fatty “plaques” in the arteries of the neck (carotid plaques). Many also have low bone density, which increases the risk of osteoporosis and fractures. We studied whether having carotid plaque is linked to poorer bone health, and whether this relationship differs between women and men. We reviewed medical records from 1,224 adults with type 2 diabetes who had both a carotid ultrasound and a bone density scan. We compared bone density results between people with and without carotid plaque, separately for women and for men. We also accounted for factors that can affect both artery disease and bone health, such as body weight and diabetes-related measures. In men, those with carotid plaque had slightly higher spine bone density, but their overall bone density categories were similar to men without plaque. In women, average bone density values looked similar between groups, but after accounting for other health factors, women with carotid plaque were more likely to fall into a worse bone-density category. Because this study looked at information from a single point in time, it cannot prove that carotid plaque causes osteoporosis. However, our findings suggest that women with type 2 diabetes who have carotid plaque may benefit from closer attention to bone health and screening for osteoporosis.

**Supplementary Information:**

The online version contains supplementary material available at 10.1186/s13293-026-00830-y.

## Introduction

Vascular calcification, particularly in the carotid arteries, is a common complication in patients with T2DM [1, 2]. It is well-established that T2DM is linked to a higher risk of cardiovascular diseases, including carotid artery stenosis, and other vascular complications [3, 4]. Carotid artery plaque, particularly when calcified, is a key marker of atherosclerosis and has been linked to higher morbidity and mortality in diabetic patients [5]. Although vascular health is linked to bone metabolism, the specific relationship between carotid artery plaque and bone metabolism in T2DM patients, particularly considering potential sex differences, remains insufficiently explored [6]. This underscores the importance of investigating this relationship to better understand its implications for bone health and early intervention in T2DM patients.

Osteoporosis, a condition marked by decreased BMD and an elevated risk of fractures, is a major concern in T2DM, particularly in postmenopausal women [7]. Notably, fracture risk in T2DM cannot be fully explained by BMD alone, highlighting the need for earlier and more comprehensive bone health assessment and risk stratification in this population [8, 9]. Previous studies have highlighted the importance of vascular health in bone metabolism, suggesting that vascular calcification could potentially contribute to bone loss [10, 11]. In particular, it has been shown that alterations in the vasculature might influence bone remodeling, with vascular calcification potentially disrupting the balance between bone formation and resorption [12].

Given the high prevalence of carotid plaque in diabetic patients, it is crucial to examine whether carotid artery plaque is linked to changes in bone metabolism, especially in postmenopausal women, to explore potential sex-specific effects. Some studies suggest that women with T2DM and vascular complications may experience more pronounced bone loss, but the exact relationship remains unclear [13]. Despite the frequent co-occurrence of atherosclerotic disease and osteoporosis in T2DM, whether carotid plaque, a clinically accessible marker of systemic atherosclerosis, is associated with altered bone metabolism and osteoporosis risk remains unclear, particularly across sexes. Bone turnover markers provide complementary information beyond BMD by reflecting dynamic processes of bone formation and resorption. Accordingly, we aimed to: (1) compare BMD and key bone turnover markers between patients with T2DM with and without carotid plaque; (2) evaluate the association between carotid plaque status and ordered osteoporosis categories (normal bone mass, osteopenia, and osteoporosis) using multivariable ordinal logistic regression; and (3) examine whether these relationships differ between men and postmenopausal women through sex-stratified analyses. By integrating carotid ultrasound–derived plaque status with DXA-derived BMD and bone turnover markers and explicitly assessing sex-specific patterns, this study provides additional evidence on bone–vascular crosstalk in older adults with T2DM and may help identify clinically relevant subgroups who could benefit from earlier bone health assessment [14].

## Materials and methods

### Study design and participants

This retrospective cross-sectional study reviewed the records of patients with T2DM hospitalized in the Department of Endocrinology and Metabolism, First Hospital of Shanxi Medical University, from May 2020 to August 2024. The study protocol was approved by the institutional ethics committee of the First Hospital of Shanxi Medical University (approval number: KYLL-2025-319), and the requirement for informed consent was waived due to the retrospective study design.

### Inclusion and exclusion criteria

**Inclusion criteria:** (1) Diagnosis of diabetes according to the American Diabetes Association (ADA) criteria [15], defined as any of the following: glycated hemoglobin (HbA1c) ≥ 6.5%; fasting blood glucose (FBG) ≥ 7.0 mmol/L; or 2-h plasma glucose ≥ 11.1 mmol/L during an oral glucose tolerance test (OGTT). (2) Eligible participants were men aged ≥ 50 years or postmenopausal women (≥ 12 consecutive months of amenorrhea without other pathological or physiological causes). (3) Patients underwent both BMD assessment using DXA and carotid artery ultrasonography during hospitalization. These criteria were applied to enrich the study population for individuals at higher risk of osteoporosis and to reduce heterogeneity related to skeletal maturation and premenopausal hormonal effects.

**Exclusion criteria:** Patients with severe hepatic dysfunction (defined as alanine aminotransferase or aspartate aminotransferase levels ≥ 3 times the upper limit of normal), severe renal dysfunction (estimated glomerular filtration rate < 30 mL/min/1.73 m² or end-stage renal disease), or severe cardiac dysfunction (New York Heart Association class III–IV heart failure or left ventricular ejection fraction < 40%) were excluded. Patients with secondary osteoporosis, including osteoporosis due to endocrine disorders (e.g., hyperthyroidism or hyperparathyroidism), chronic kidney disease–mineral and bone disorder, or medication-induced osteoporosis, were also excluded. In addition, patients receiving medications known to affect bone metabolism, such as long-term systemic glucocorticoids, anti-osteoporotic agents (e.g., bisphosphonates or denosumab), anticonvulsants, or aromatase inhibitors, were excluded.

### Sample size calculation

**Fig. 1:**
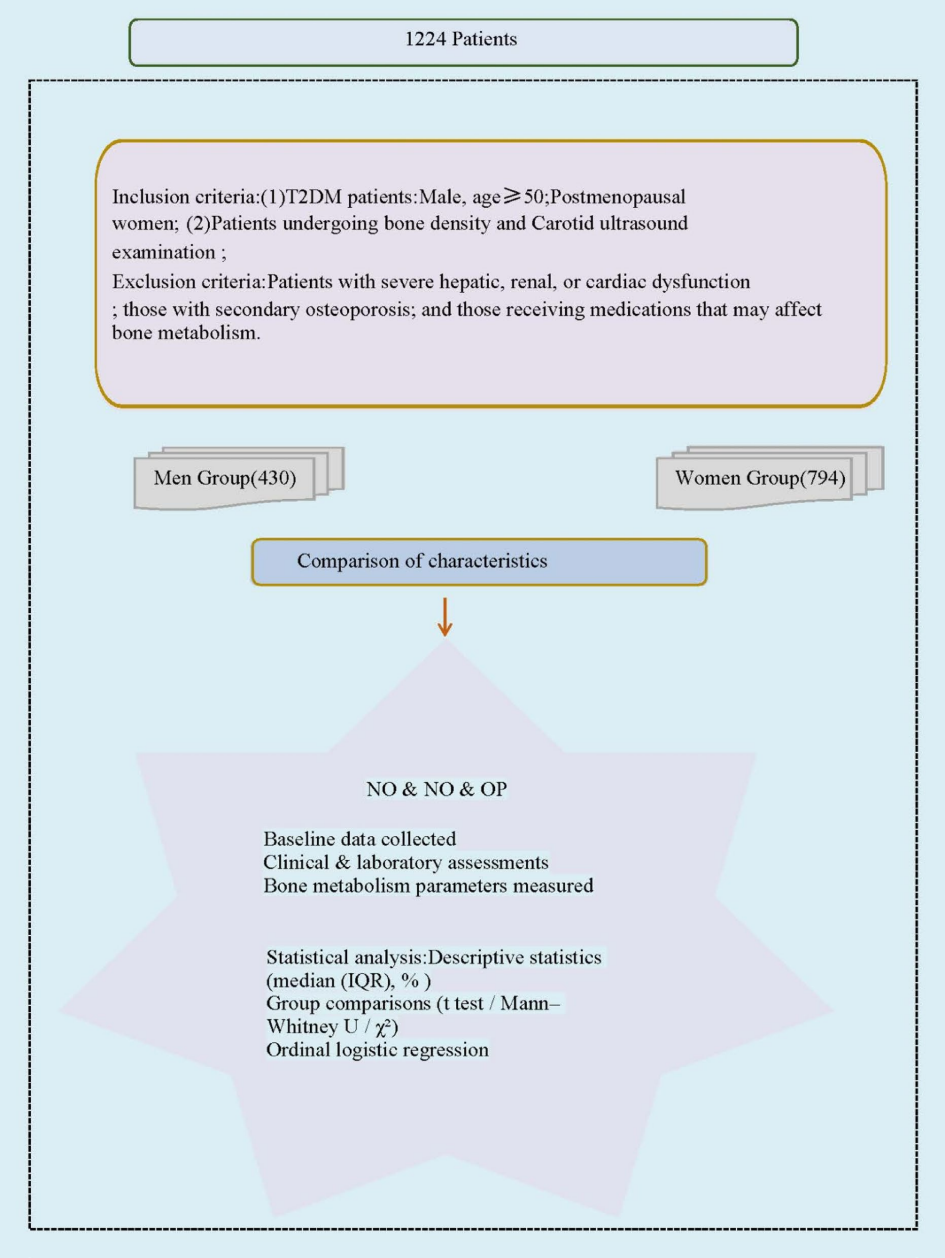
Flow chart

Sample size was estimated using the standard formula for cross-sectional studies:

$$\:N=\frac{{Z}_{1-{\upalpha\:}/2}^{2}p(1-p)}{{d}^{2}}$$

where $\:{Z}_{1-\frac{\alpha\:}{2}}\:$is the standard normal deviate corresponding to the two-sided significance level$\:\alpha\:$, $\:p\:$is the expected prevalence, and $\:d\:$is the desired precision. We used $\:\alpha\:=0.05$(i.e.,$\:{Z}_{1-\frac{\alpha\:}{2}}=1.96$) and$\:d=0.05$. Based on assumed prevalence of $\:p=0.144$for men and $\:p=0.207$for women, the minimum required sample sizes were 190 men and 252 women, respectively. The final study population included 430 men and 794 women, exceeding the calculated minimum requirements and yielding achieved precisions of approximately 3.3% for men and 3.0% for women (Fig. 1).

### Bone mineral density and carotid artery ultrasonography

Lumbar spine BMD (g/cm²) at L1–L4 was measured using a Hologic Discovery Wi DXA system (Hologic Inc., Marlborough, MA, USA; APEX software v13.4.2.5). The densitometer underwent daily calibration and quality control using a manufacturer-supplied phantom. The in vivo precision error (coefficient of variation, CV%) for lumbar spine BMD in our center was 0.8%, based on repeated measurements. T-scores were calculated using the Hologic sex-specific reference database (men: Hologic male reference; women: Hologic female reference).

Osteoporosis status was defined according to World Health Organization (WHO) criteria based on lumbar spine (L1–L4) T-scores: normal bone mass (NO; T-score > − 1.0), osteopenia (ON; −2.5 < T-score ≤ − 1.0), and osteoporosis (OP; T-score ≤ − 2.5). All lumbar spine scans were visually inspected for artifacts (e.g., vertebral fractures, osteophytes, and abdominal aortic calcification), and scans with significant artifacts that could affect BMD were excluded. If a vertebra was affected by artifacts, it was excluded and the L1–L4 result was calculated from the remaining evaluable vertebrae (at least two vertebrae required).

Carotid artery ultrasonography was performed to evaluate carotid atherosclerosis using either a GE Healthcare LOGIQ E11 ultrasound system equipped with an L2–9 linear-array transducer (2–9 MHz) or a SIEMENS ACUSON Sequoia ultrasound system equipped with a 10L4 linear-array transducer (4–7.3 MHz). Bilateral carotid arteries were systematically examined, including the common carotid artery (CCA), carotid bifurcation, and internal carotid artery (ICA). Carotid plaque was defined as a focal structure that encroaches into the arterial lumen by at least 0.5 mm or 50% of the surrounding intima–media thickness (IMT), or has a thickness > 1.5 mm measured from the media–adventitia interface to the intima–lumen interface, in accordance with the Mannheim consensus. Based on plaque presence, participants were classified into two groups: CP (carotid plaque present) and NCP (no carotid plaque). All examinations were performed by trained sonographers who were blinded to participants’ clinical information and followed a standardized scanning protocol. Images with uncertain plaque identification were independently reviewed by a second sonographer, and discrepancies were resolved by consensus. IMT was measured, but it was not included in the present analysis, as the primary objective of this study was to evaluate plaque presence rather than IMT.

### Laboratory measurements

These bone turnover markers were included because they provide complementary information beyond BMD and may reflect sex-specific differences in bone remodeling in T2DM. Participants fasted for at least 12 h before blood collection. Samples were collected in the morning to minimize diurnal variation, and laboratory analyses were completed within 2 h after sampling.

Serum bone turnover markers and related hormones, including total procollagen type I N-terminal propeptide (PINP), β-isomerized C-terminal telopeptide of type I collagen (β-CTX), parathyroid hormone (PTH), and N-MID osteocalcin (bone Gla protein, BGP), were measured by electrochemiluminescence immunoassay (ECLIA) on Roche cobas e411/e601/e602 immunoassay analyzers using the corresponding Elecsys reagent kits (Roche Diagnostics). PINP, β-CTX, and PTH were determined using sandwich ECLIA. The intra-assay CV for PINP ranged from 1.2% to 3.2%, and the inter-assay (intermediate precision) CV ranged from 1.7% to 4.1%. For β-CTX, the intra-assay CV ranged from 1.2% to 6.5%, and the inter-assay CV ranged from 1.4% to 10.7%. For PTH, the intra-assay CV ranged from 1.1% to 2.0%, and the inter-assay CV ranged from 2.5% to 3.4%. Serum N-MID osteocalcin (BGP) was quantified using the Roche e601 N-MID osteocalcin assay, with an intra- and inter-assay CV of 4.49%.

Serum 25-hydroxyvitamin D (25(OH) D) was measured using the Elecsys 25(OH) D total assay based on a competitive ECLIA method. The intra-assay CV ranged from 1.3% to 6.3%, and the inter-assay CV ranged from 1.5% to 8.9%.

Serum lipid profiles, including total cholesterol (TC), triglycerides (TG), high-density lipoprotein cholesterol (HDL-C), and low-density lipoprotein cholesterol (LDL-C), were measured using standard enzymatic methods on a Beckman AU5800 analyzer. Calibration was performed every two weeks using certified reference materials. All lipid parameters were expressed in mmol/L.

### Statistical analysis

All statistical analyses were performed using SPSS version 25.0 (IBM Corp., Armonk, NY, USA). Continuous variables were summarized as medians with interquartile ranges (IQR), and categorical variables were presented as frequencies and percentages. Data distribution was assessed using the Shapiro–Wilk test. Comparisons between groups (CP vs. NCP) were conducted using the Mann–Whitney U test for continuous variables and the chi-square (χ²) test or Fisher’s exact test, as appropriate, for categorical variables.

To investigate the association between carotid plaque and osteoporosis status, an ordinal logistic regression model (proportional odds model) was fitted with osteoporosis status as the dependent variable (NO/ON/OP). Results are presented as ORs with 95% CIs and corresponding P values. Covariates were selected a priori based on clinical relevance and previous evidence linking metabolic and vascular risk factors with both carotid atherosclerosis and osteoporosis/bone mineral density. Covariate selection and model building followed standard methodological recommendations [16].

Multivariable models were constructed with stepwise adjustment: Model 1 adjusted for duration of diabetes and body mass index (BMI); Model 2 further adjusted for lipid parameters (TC, TG, HDL-C, and LDL-C); and Model 3 additionally adjusted for glycemic indices (FBG and HbA1c). Sex-stratified analyses were performed separately in men and postmenopausal women. Given the significant between-group difference in age, sex-stratified sensitivity analyses with additional adjustment for age were conducted, and the estimates were not materially changed (Supplementary Table S1).

The proportional odds (parallel lines) assumption was assessed using the Test of Parallel Lines, with *P* > 0.05 indicating no violation. When this assumption was violated (*P* < 0.05), multinomial logistic regression was additionally conducted as a sensitivity analysis for the affected models, and the corresponding results are presented in Supplementary Table S2.

To evaluate potential synergy between 25(OH) D and calcium (Ca), we tested a 25(OH) D × Ca interaction term using lumbar spine BMD as a continuous outcome. 25(OH) D and Ca were standardized (Z-scores), and the interaction term was constructed as Z(25(OH) D) × Z(Ca). Sex-stratified models were fitted in men and postmenopausal women. In the combined sample, a three-way interaction term (Sex × Z(25(OH) D) × Z(Ca); Sex coded as 0 = men and 1 = women) was additionally included to formally test whether the interaction differed by sex. Multicollinearity was assessed using variance inflation factors (VIF) (Supplementary Tables S3–S6).

To address potential inflation of lumbar spine DXA measures due to abdominal aortic calcification and/or degenerative changes, vertebra-level sensitivity analyses were performed by recalculating lumbar spine BMD using the mean of L1–L2 and the mean of L1–L3, and repeating group comparisons (Supplementary Table S7).

All statistical tests were two-sided, and a P value < 0.05 was considered statistically significant.

## Results

### Clinical characteristics of patients

This study included a total of 1,224 patients with T2DM, consisting of 794 women and 430 men. When stratified by carotid plaque status, patients in the plaque group were significantly older and had a longer duration of diabetes compared with those without plaque in both sexes (all *P* < 0.001). Among women, patients with plaque had higher systolic blood pressure (SBP) (*P* = 0.001) and lower diastolic blood pressure (DBP) (*P* = 0.017), as well as higher levels of FBG and HbA1c (both *P* < 0.001). In men, the plaque group showed significantly lower HDL-C levels (*P* = 0.023) and higher FBG and HbA1c levels (both *P* ≤ 0.001). No significant differences were observed in BMI, TC, TG, LDL-C, or serum creatinine between groups (Table 1).

### Bone metabolism markers

Bone metabolism markers, including osteocalcin, PINP, and β-CTX, were selected because they reflect bone turnover dynamics. Osteocalcin and PINP are commonly used markers of bone formation, whereas β-CTX reflects bone resorption and typically increases when bone breakdown is accelerated.

In women, the plaque group had significantly lower PTH levels (*P* = 0.028) and lower β-CTX levels (*P* = 0.003). In men, the plaque group had significantly lower osteocalcin (*P* < 0.001) and PINP (*P* = 0.014), along with lower β-CTX levels (*P* < 0.001). Taken together, the concurrent reduction in formation markers (osteocalcin, PINP) and the resorption marker (β-CTX) suggests a low–bone-turnover (“adynamic”) phenotype, rather than isolated high bone resorption. No significant differences were observed in Ca, phosphorus (P), magnesium (Mg), or 25(OH) D levels between groups (Table 2).

**Table 1:** Clinical characteristics of the study patients stratified by sex and plaque

	Women					Men				
	Total(*n*=794)	Without plaque(*n*=329)	Plaque(*n*=465)	*P* Value	Effect size (*r*/Cramér’s V)	Total(*n*=430)	Without plaque(*n*=112)	Plaque(*n*=318)	*P* Value	Effect size (*r*/Cramér’s V)
Age(years)	**64(58**,**70)**	**60(55**,**67)**	**67(60**,**72)**	**<0.001**	0.302	**63(58**,**69)**	**60(56**,**66)**	**64(59**,**70)**	**<0.001**	0.185
BMI(kg/m²)	23.5(21.47,25.96)	23.73(21.5,25.71)	23.44(21.3,26.17)	0.679	0.018	24.21(22.29,26.21)	24.12(21.75,25.78)	24.21(22.49,26.33)	0.190	0.063
SBP(mmHg)	**132(121**,**144)**	**130(119**,**142)**	**135(122**,**145)**	**0.001**	0.119	130(120,142)	130(116.25,141)	130(121,143.5)	0.122	0.075
DBP(mmHg)	79(71,87)	80(72,90)	**78(70**,**85)**	**0.017**	0.085	80(73,88)	81.5(72.25,88)	80(73,88)	0.908	0.006
Pulse(beats/min)	80(75,86)	80(75,87)	80(76,86)	0.78	0.013	80(75,86)	80(74,88)	80(75,86)	0.740	0.016
Diabetes Duration(years)	**6(1**,**15)**	**1(0**,**10)**	**10(2**,**19)**	**<0.001**	0.327	**10(1**,**19.5)**	**4(1**,**12)**	**10(2**,**20)**	**<0.001**	0.251
TC(mmol/L)	4.59(3.83,5.37)	4.73(4.0,5.35)	4.54(3.79,5.44)	0.409	0.029	4.18(3.44,4.95)	4.09(3.42,4.9)	4.24(3.46,4.98)	0.665	0.021
TG(mmol/L)	1.44(1.02,2.0)	1.43(1.02,1.95)	1.47(1.02,2.05)	0.454	0.027	1.29(0.94,1.9)	1.17(0.91,1.78)	1.33(0.96,1.95)	0.126	0.074
HDL(mmol/L)	1.2(1.03,1.37)	1.2(1.05,1.4)	1.19(1.01,1.36)	0.104	0.058	**1.06(0.91**,**1.27)**	**1.13(0.93**,**1.37)**	**1.04(0.90**,**1.25)**	**0.023**	**0.110**
LDL(mmol/L)	2.87(2.29,3.44)	2.94(2.36,3.43)	2.82(2.25,3.45)	0.349	0.033	2.64(2.07,3.15)	2.56(2.07,3.20)	2.69(2.08,3.16)	0.680	0.020
Cr(μmol/L)	55.55(48,64.7)	55.1(48.2,64.4)	55.7(47.8,64.8)	0.580	0.020	68.1(59.5,78.35)	65.1(58.75,74.75)	68.8(60,81.5)	0.051	0.094
FBG(mmol/L)	**6.10 (5.00**,** 8.14)**	**5.61(4.98**,**7.5)**	**6.6(5.18**,**8.38)**	**<0.001**	0.151	**6.85(5.23**,**8.61)**	**5.83(4.67**,**7.80)**	**7.0(5.45**,**8.70)**	**0.001**	**0.167**
HbA1c(%)	**7.36(6.0**,**9.16)**	**6.61(5.72**,**8.32)**	**7.84(6.43**,**9.5)**	**<0.001**	0.229	**7.64(6.32**,**9.33)**	**6.48(5.46**,**8.99)**	**7.85(6.83**,**9.52)**	**<0.001**	**0.239**
Bone status										
Normal(n, %)	**-**	85(25.84)	97(20.86)	0.239	0.060	**-**	44(39.29)	174(54.72)	0.019	0.136
Osteopenia(n, %)	**-**	106(32.22)	166(35.70)	0.239	0.060	**-**	43(38.39)	90(28.30)	0.019	0.136
Osteoporosis (n, %)	-	138(41.95)	202(43.44)	0.239	0.060	-	25(22.32)	54(16.98)	0.019	0.136

Values are presented as median (IQR, 25th–75th percentile) unless otherwise indicated. Continuous variables were compared using the Mann–Whitney U test, and effect size is reported as r = |Z|/√N. Bone status (NO/ON/OP) was compared using Pearson χ², and effect size is reported as Cramér’s V. Statistically significant P values (*P* < 0.05) are highlighted in bold

Abbreviations: BMI: Body Mass Index; SBP: systolic blood pressure; DBP: diastolic blood pressure; TC: Total Cholesterol; TG: triglycerides; HDL: high-density lipoprotein cholesterol; LDL:

Low-density lipoprotein cholesterol; Cr: Creatinine; FBG: fasting blood glucose; HbA1c: Glycated hemoglobin

### Differences in bone mineral density grouped by sex and plaque

**Fig. 2:**
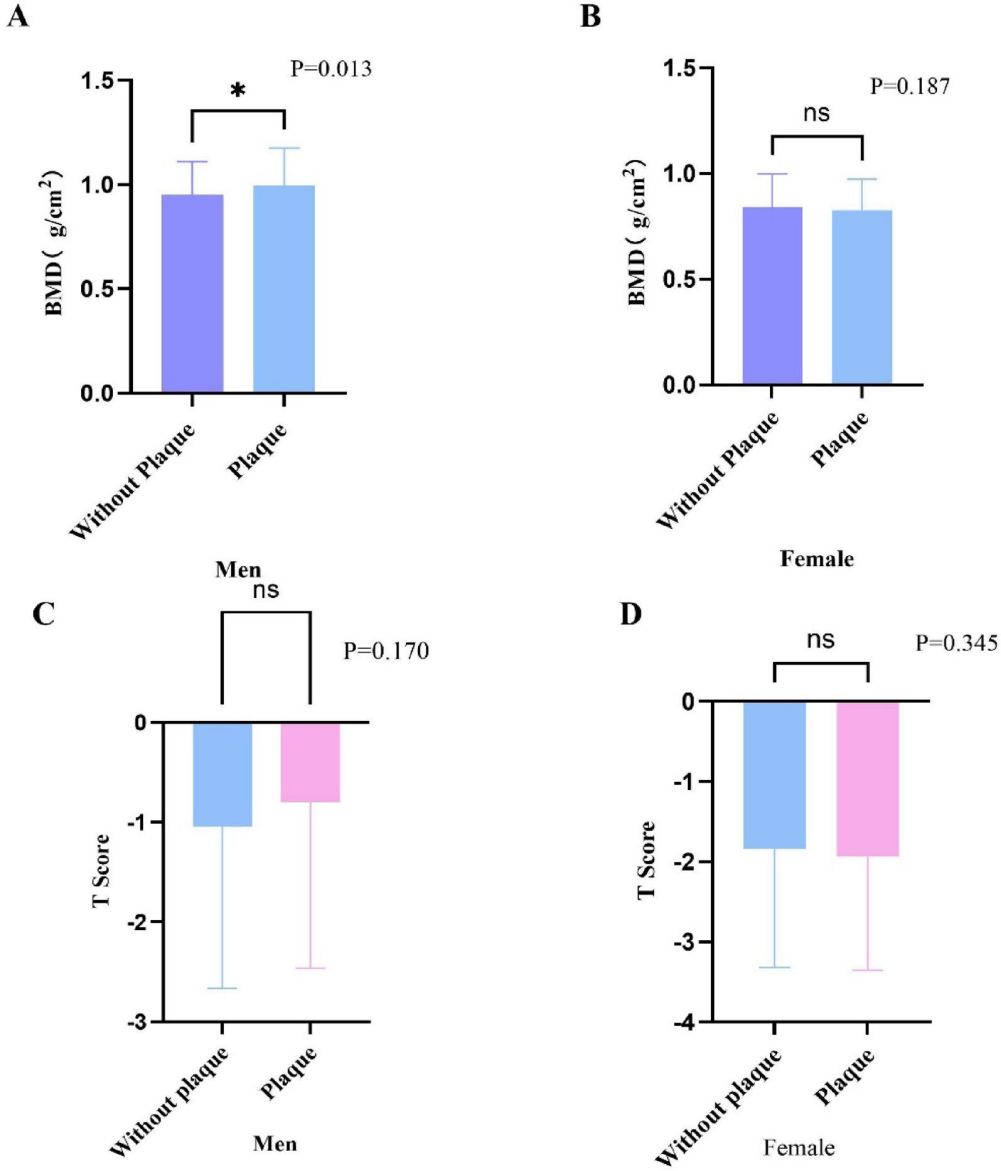
Comparison of BMD and T-Scores between Patients with and without Carotid Plaques, Stratified by Sex. The left panel (**A**) shows BMD values (g/cm²) in men, with a significant difference observed between those with and without carotid plaques (*P = 0.013*). The right panel (**B**) illustrates BMD in women, where no significant difference was found between the two groups (*P = 0.187*). The lower panels (**C** and **D**) display T-scores for men and women, respectively. For men (**C**), there is no significant difference in T-scores between the two groups (*P = 0.170*). Similarly, for women (**D**), no significant difference in T-scores was observed (*P = 0.345*)

In men, the prevalence of osteoporosis did not differ significantly between the plaque and non-plaque groups (Table 1). Accordingly, lumbar spine T-scores did not differ significantly (Fig. 2C). However, men with plaque had a significantly higher lumbar spine BMD compared with those without plaque (*P* = 0.013, Fig. 2A).

**Table 2:** Bone turnover markers in different groups

	Women					Men				
	Total(*n*=794)	Without plaque(*n*=329)	Plaque(*n*=465)	*P* Value	Effect size (*r*/Cramér’s V)	Total(*n*=430)	Without plaque(*n*=112)	Plaque(*n*=318)	*P* Value	Effect size (*r*)
Ca(mmol/L)	2.26(2.19,2.34)	2.27(2.18,2.35)	2.26(2.2,2.33)	0.495	0.024	2.25(2.17,2.33)	2.23(2.16,2.32)	2.25(2.18,2.33)	0.139	0.071
P(mmol/L)	1.23(1.1,1.36)	1.23(1.08,1.34)	1.24(1.11,1.37)	0.141	0.052	1.16(1.06,1.3)	1.18(1.08,1.33)	1.15(1.04,1.3)	0.206	0.061
Mg(mmol/L)	0.87(0.82,0.929)	0.87(0.83,0.92)	0.87(0.81,0.92)	0.351	0.033	0.85(0.80,0.90)	0.86(0.79,0.90)	0.85(0.81,0.90)	0.892	0.007
25(OH)D(ng/ml)	35.55(25.81,49.08)	35.89(24.16,49.38)	35.5(26.28,48.89)	0.691	0.014	45.67(31.43,62.11)	45.44(32.96,66.52)	46.17(30.62,61.4)	0.546	0.029
Osteocalcin(ng/ml)	15.44(10.87,24.99)	16.24(10.77,27.07)	14.83(10.94,23.45)	0.111	0.057	**12.79(8.97**,**18.71)**	**14.95(11.4**,**22.41)**	**11.98(8.6**,**17.83)**	**<0.001**	0.187
PTH(pg/ml)	**33.2(23.66**,**45.85)**	**35.01(24.77**,**47.86)**	**32.14(23.18**,**44.52)**	**0.028**	0.078	33.1(24.21,44.54)	33.92(24.12,49.37)	33.01(24.21,44.23)	0.576	0.027
PINP(ng/ml)	45.64(29.98,65.85)	46.83(29.06,71.46)	44.95(30.24,62.65)	0.198	0.046	**38.38(26.36**,**54.95)**	**45.12(28.27**,**64.85)**	**36.88(26.22**,**52.5)**	**0.014**	0.119
β-CTX(ng/ml)	**430.8(290.65**,**670.32)**	**470.9(314.8**,**751.6)**	**410.8(281.1**,**634.6)**	**0.003**	0.106	**398.4(262.2**,**568.3)**	**465.6(311.35**,**782.98)**	**378.5(254.25**,**519.55)**	**<0.001**	0.173

Values are presented as median (IQR, 25th–75th percentile). Continuous variables were compared using the Mann–Whitney U test, and effect size is reported as r = |Z|/√N. Statistically significant P values (*P* < 0.05) are highlighted in bold

Abbreviations: Ca: serum calcium; P:serum phosphorus; Mg: serum magnesium;25(OH)D:25 hydroxyvitamin D; PTH: parathyroid hormone; PINP: Procollagen I N-Terminal Propeptide;

β-CTX:β-Collagen Degradation Products;

In women, the prevalence of osteoporosis was similar between the two groups (Table 1). Consistently, both lumbar spine BMD and T-scores showed no significant differences between women with and without plaque (Fig. 2B and D).

Although a significant difference in lumbar spine BMD was observed in unadjusted comparisons, this finding did not translate into a higher prevalence of osteoporosis or lower T-scores, suggesting that the observed BMD difference may be influenced by factors other than true skeletal strength, such as degenerative changes or body composition–related effects on DXA measurements. To address potential DXA inflation from abdominal aortic calcification and/or degenerative changes, vertebra-level sensitivity analyses were conducted using mean BMD of L1–L2 and L1–L3; the higher BMD in men with plaque persisted (*p* = 0.017 for both), whereas no differences were observed in women (all *p* > 0.26) (Supplementary Table S7).

We additionally tested whether 25(OH) D and Ca exerted synergistic effects on lumbar spine BMD by including a standardized interaction term (Z(25(OH) D) × Z(Ca)) in multivariable models. The 25(OH) D × Ca interaction was not significant in sex-stratified analyses (men: ΔR² = 0.000, *p* = 0.959; women: ΔR² = 0.000, *p* = 0.651). In the combined sample, neither the two-way interaction (*p* = 0.312) nor the Sex × 25(OH) D × Ca three-way interaction (ΔR² = 0.000, *p* = 0.809) was significant (Supplementary Tables S3–S6).

### Ordinal logistic regression analysis of carotid plaques and osteoporosis

In men, no significant associations between carotid plaques and osteoporosis were observed across all three models. In Model 1, adjusting for diabetes duration and BMI, the OR was 0.729 (95% CI: 0.476–1.118, *P* = 0.147). This trend remained non-significant in subsequent models, with the OR in Model 2 at 0.773 (95% CI: 0.502–1.189, *P* = 0.241), and in Model 3 at 0.791 (95% CI: 0.511–1.224, *P* = 0.292).

In contrast, women consistently showed a significant positive association between carotid plaques and osteoporosis across all models. In Model 1, the OR was 1.395 (95% CI: 1.052–1.850, *P* = 0.021). After adjusting for lipid parameters in Model 2, the OR increased to 1.448 (95% CI: 1.090–1.924, *P* = 0.011), and further adjustment in Model 3 resulted in an OR of 1.496 (95% CI: 1.124–1.992, *P* = 0.006).

In sex-stratified sensitivity analyses with additional adjustment for age, the association between carotid plaque and osteoporosis status was not materially changed in men or postmenopausal women (Supplementary Table S1). In postmenopausal women, the proportional odds assumption was violated in age-adjusted Models 2 and 3; therefore, multinomial logistic regression was performed as a sensitivity analysis. The multinomial results showed no significant association between carotid plaque and either osteopenia or osteoporosis compared with normal bone mass, and were consistent with the findings from the ordinal models (Supplementary Table S2).

**Fig. 3:**
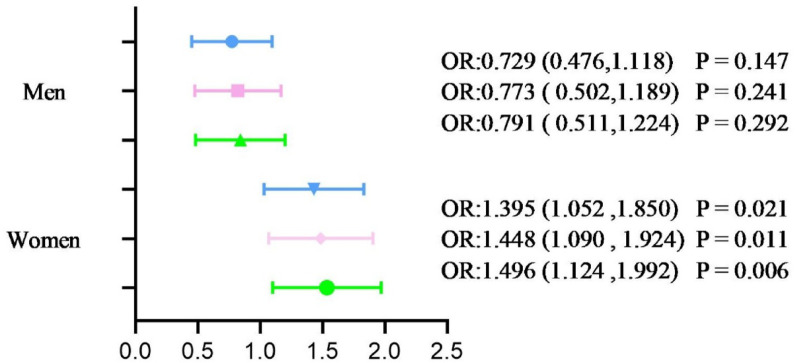
Ordinal Logistic Regression Analysis of Factors Associated with Osteoporosis. Dependent variables included normal BMD, osteopenia, and osteoporosis. Independent variables included carotid plaque presence and various potential confounding factors. Model 1: Adjusted for BMI and duration of T2DM. Model 2: Further adjusted for TC, TG, HDL, and LDL. Model 3: Additionally adjusted for FBG and HbA1c. OR and 95% CI are shown for each model, with significant associations indicated for women (*P < 0.05*) but not for men

Taken together, these findings indicate a stable sex-specific pattern, with a positive association between carotid plaques and osteoporosis observed in women but not in men. This difference may reflect sex-specific biological mechanisms, such as hormonal influences on both bone metabolism and vascular health (Fig. 3).

## Discussion

### Principal findings

This study demonstrated that, after stepwise adjustment for multiple cardiometabolic factors, the presence of carotid plaque on ultrasound was significantly associated with worse osteoporosis status in women with T2DM, whereas no such association was observed in men. This sex-specific pattern suggests that the bone–vascular relationship in T2DM is not uniform across sexes and supports the hypothesis that the association may depend on both carotid plaque status and sex. Importantly, the direction and magnitude of the association in women remained stable across increasingly adjusted models, and sensitivity analyses using multinomial logistic regression—performed when the proportional odds assumption was violated—yielded consistent results. Collectively, these findings extend existing evidence on bone–vascular interplay by highlighting a potentially clinically relevant subgroup, namely women with T2DM and carotid plaques, who may warrant closer osteoporosis risk assessment.

### Clarification of terminology

In this study, plaque characteristics (including plaque composition) refer to the relative tissue components within plaques (e.g., predominantly lipid-rich/necrotic versus more fibrous or calcified plaques). Calcification characteristics refer to the presence and extent of calcium deposition within plaques and/or the vascular wall.

### Interpretation of sex-specific patterns and confounding

An apparent discrepancy was observed between unadjusted and adjusted analyses. Unadjusted comparisons of continuous bone measures showed a significant association between lumbar spine BMD and carotid plaque in men, whereas no such correlation was detected in women. In contrast, multivariable models demonstrated a significant association between carotid plaque and osteoporosis status in women but not in men. These differences likely reflect the influence of confounding factors, particularly age and body mass index, which can substantially affect both bone density and vascular pathology. Multivariable modeling therefore provides a more appropriate estimate of the independent association between carotid plaque and osteoporosis.

In men, the higher unadjusted lumbar spine BMD observed in those with carotid plaque did not translate into differences in T-scores or osteoporosis prevalence and was no longer evident after multivariable adjustment. Moreover, lumbar spine DXA measurements are particularly susceptible to degenerative changes such as osteophyte formation, endplate sclerosis, and adjacent vascular calcification, which may artifactually elevate BMD estimates. Therefore, this finding is unlikely to reflect true improvements in bone strength and should be interpreted cautiously.

### Bone turnover profile

With respect to bone turnover, the biomarker profile suggested a suppressed remodeling milieu. In men, carotid plaques were associated with lower formation markers (osteocalcin and PINP) together with reduced β-CTX, indicating a more global low–bone-turnover pattern. In women, carotid plaques were mainly associated with lower β-CTX, whereas osteocalcin and PINP did not differ significantly between plaque groups, suggesting that the plaque-related signal may predominantly involve the resorption/remodeling arm or reflect a constrained remodeling capacity not fully captured by the available formation markers. This pattern is more consistent with a low–bone-turnover (adynamic) phenotype rather than a state of high bone resorption [17, 18]. Low bone turnover—particularly reduced bone formation—is a recognized feature of diabetic bone disease and may contribute to impaired bone quality and increased fracture risk even when BMD is not markedly reduced [18, 19].

### Comparison with previous studies

Although research on the relationship between carotid plaques and bone health remains limited, accumulating evidence supports a link between atherosclerotic burden and skeletal deterioration [20, 21]. For instance, a cross-sectional study of individuals aged ≥ 50 in Vietnam found an association between elevated carotid IMT and bone density levels, suggesting an inherent link between osteoporosis and atherosclerosis [22]. Additionally, studies have shown that in women without clinical signs of atherosclerosis, carotid plaques are closely associated with decreased femoral neck bone density, and coronary artery calcification index is independently related to systemic bone loss [23]. A study by Choi SH et al. found that in middle-aged women, an increased burden of atherosclerotic plaques was independently associated with low bone density. This further supports the potential connection between atherosclerosis and bone metabolism, suggesting that plaques may serve as important indicators of bone loss [24]. In African American individuals with T2DM, an inverse relationship between lumbar spine BMD and calcified arterial plaques has also been observed [25, 26].

### Potential mechanisms (hypothesis-generating; correlation ≠ causation)

Macrovascular disease may further aggravate this low-formation/low-remodeling milieu through systemic endothelial dysfunction, oxidative stress, chronic low-grade inflammation, and impaired skeletal perfusion, thereby reinforcing osteoblast suppression and blunting adaptive remodeling. However, because our study was cross-sectional and mechanistic biomarkers (e.g., sex hormones, inflammatory cytokines, and bone–vascular pathway markers) were not measured, these mechanistic interpretations remain hypothesis-generating rather than being directly demonstrated by our data, and the observed associations should not be interpreted as causal.

These findings suggest that the observed sex differences in the association between carotid plaques and osteoporosis may be driven by shared yet sex-specific pathological mechanisms, including hormonal regulation, inflammatory responses, and disturbances in bone–vascular signaling pathways [27]. However, the present study did not include direct measurements of key mechanistic biomarkers (e.g., circulating sex hormones, inflammatory cytokines such as interleukin-6 (IL-6) and tumor necrosis factor-alpha (TNF-α), oxidative stress markers, receptor activator of nuclear factor-κB ligand (RANKL)/receptor activator of nuclear factor-κB (RANK)/osteoprotegerin (OPG)-related indices, or osteocalcin carboxylation status). Accordingly, the following mechanistic considerations are hypothesis-generating and based primarily on prior literature rather than being demonstrated by our data. Estrogen receptors have been found in both bone (osteoclasts and osteoblasts) and blood vessels (endothelial and smooth muscle cells), suggesting that estrogen may directly influence bone metabolism and have clinical significance for atherosclerosis [28, 29]. The decline in estrogen levels post-menopause promotes inflammation and bone resorption, thereby increasing the risk of osteoporosis and vascular calcification [30, 31]. Inflammatory factors, including TNF-α and IL-6, and the RANKL/RANK/OPG signaling pathway play significant roles in both bone loss and the progression of atherosclerosis [32, 33]. Additionally, disturbances in calcium-phosphorus metabolism and chronic oxidative stress may concurrently drive both bone loss and vascular stiffening [32, 34].

### Future directions

Future studies should incorporate mechanistic biomarkers (e.g., sex hormones, inflammatory cytokines, and RANKL/RANK/OPG-related markers) and include individuals without T2DM as comparators to better delineate sex-specific bone–vascular pathways. Longitudinal designs with standardized capture of fractures and other clinically meaningful outcomes are also needed to clarify temporal relationships and clinical relevance.

### Strengths

Several strengths of this study should be highlighted. Unlike previous studies that focused solely on overall vascular health, this study specifically examined carotid plaques as a clinically accessible marker of bone density loss, offering new insights into the intersection of cardiovascular and skeletal health. It is noteworthy that diabetes-related microvascular complications, such as retinopathy and nephropathy, have also been shown to be associated with low bone density [35, 36]. The mechanisms may differ from those of macrovascular disease: the former not only provides blood supply and nutrition to bone tissue but also participates in the regulation of bone metabolism, while macrovascular disease primarily affects the skeleton through perfusion [37].

### Limitations

It should be noted that this study, based on carotid ultrasound, only assessed the presence or absence of plaques without further analyzing the composition and calcification characteristics (e.g., lipid-rich versus calcified plaques). This limitation somewhat restricts a deeper understanding of the relationship between vascular calcification and osteoporosis. Additionally, this study only evaluated lumbar spine bone density and did not analyze bone mass in other sites, such as the hip and femoral neck, thus failing to fully reveal the relationship between arterial calcification in different regions and bone density. Previous studies have suggested that lower limb arterial calcification more directly affects femoral neck and hip bone mass [38], while carotid plaques may indicate systemic bone loss. This aspect warrants further exploration in future research.

This study has several limitations. First, due to the retrospective cross-sectional design, the analysis is subject to potential selection bias and residual or unmeasured confounding, and temporality cannot be established; therefore, the observed associations should not be interpreted as causal. Reverse causation cannot be excluded, and a shared unmeasured factor may partially contribute to both carotid plaque and impaired bone health. Future longitudinal, prospective studies with repeated assessments are warranted to clarify directionality and temporality and to evaluate dynamic changes in vascular and skeletal health. The discrepancy between the significant unadjusted correlations and the null associations in adjusted models may reflect confounding by metabolic factors and residual confounding due to unmeasured lifestyle and medication-related variables.

Second, bone health assessment was based primarily on lumbar spine DXA measures (BMD and T-scores) and categorized osteoporosis status. Additional clinically relevant endpoints, such as incident fractures, as well as DXA measurements at multiple skeletal sites (e.g., total hip and femoral neck), should be incorporated in future work to improve comprehensiveness and clinical interpretability [39].

Third, data on fracture outcomes and key lifestyle factors, including physical activity and dietary or nutritional status, were not available, information on medication use was also limited, which may have introduced residual confounding. In addition, mechanistic biomarkers—including circulating sex hormones, inflammatory markers (e.g., IL-6 and TNF-α), and osteocalcin carboxylation status—were not collected; therefore, we could not directly assess whether endocrine or inflammatory pathways mediate or modify the observed sex-specific associations. Moreover, vitamin K status was not measured in this study. Given its biological interplay with 25(OH) D and Ca in bone and vascular health, we could not evaluate the broader 25(OH) D–Ca–vitamin K axis. Although we tested the 25(OH) D× Ca interaction on lumbar spine BMD and found no evidence of synergy or sex-specific modification (Supplementary Tables S2–S5), future studies incorporating vitamin K biomarkers (e.g., circulating vitamin K forms and/or functional markers such as undercarboxylated osteocalcin) are warranted.

Fourth, carotid ultrasound, while widely used for plaque detection, primarily identifies the presence of plaques and cannot comprehensively characterize plaque composition or stability. Imaging modalities capable of assessing plaque vulnerability, such as high-resolution magnetic resonance imaging (MRI) or CT angiography (CTA), may strengthen future investigations. In addition, the studied subgroups were not optimally matched in size, with fewer men than women, which may have limited statistical power and should be considered when interpreting the sex-specific results.

Fifth, the study population consisted of individuals with type 2 diabetes (and from a single center/region, if applicable), which may limit the generalizability of our findings to non-diabetic populations or other clinical settings. Therefore, the results should be extrapolated with caution. Future multicenter studies in more diverse populations are warranted to validate these sex-specific associations.

## Conclusion

In summary, this study found a sex-specific association between carotid plaque and osteoporosis severity in patients with T2DM, with a significant adjusted association observed in women but not in men. Because carotid ultrasonography is widely performed in routine cardiovascular assessment, the presence of carotid plaque may help identify women with T2DM who could benefit from earlier or more intensive osteoporosis evaluation [40]. While these findings suggest that carotid plaque may be a potential indicator of poorer skeletal status in women with T2DM, the interpretation is limited by the retrospective cross-sectional design and the lack of key confounders (including lifestyle factors, central adiposity measures, medication exposure/adherence, and fracture outcomes). Mechanistic interpretations (including hormonal pathways) are limited because sex hormone levels were not assessed in the present study. Therefore, our results should not be construed as causal, and clinical implications should be confirmed in future prospective longitudinal studies that incorporate fracture endpoints and more comprehensive covariate assessment.

## Supplementary Information

Supplementary Material 1

## Data Availability

The datasets used and analyzed during the current study are available from the corresponding author on reasonable request.

## Abbreviations

T2DM: Type 2 diabetes mellitus
BMD: Bone mineral density
BMI: Body Mass Index
SBP: Systolic blood pressure
DBP: Diastolic blood pressure
TC: Total Cholesterol
TG: Triglycerides
HDL-C: High-density lipoprotein cholesterol
LDL-C: Low-density lipoprotein cholesterol
Cr: Creatinine
Ca: Serum calcium
P: Serum phosphorus
Mg: Serum magnesium
25(OH) D: 25 hydroxyvitamin D
BGP: Bone Gla-Protein
PTH: Parathyroid hormone
PINP: Procollagen I N-Terminal Propeptide
β-CTX: β-Collagen Degradation Products
FBG: Fasting blood glucose
HbA1c: Glycated hemoglobin
RANKL: Receptor activator of nuclear factor-κB ligand
RANK: Receptor activator of nuclear factor-κB
OPG: Osteoprotegerin; IL-6:interleukin-6
TNF-α: Tumor necrosis factor-alpha
IMT: Intima–media thickness
OR: Odds ratio
95% CI: 95% confidence interval
CV%: Coefficient of variation
IQR: Interquartile ranges
