# Supplementary material for: Sex-specific associations between carotid plaque and bone mineral density in patients with type 2 diabetes: a retrospective cross-sectional study
Source: Biol Sex Differ. 2026 Jan 28;17:35. doi: 10.1186/s13293-026-00830-y (PMC12922300; doi:10.1186/s13293-026-00830-y)
Supplement: Supplementary file 1 — Supplementary Material 1 [file 13293_2026_830_MOESM1_ESM.docx]

*Title Page*

**Sex-Specific Associations Between Carotid Plaque and Bone Mineral Density in Patients With Type 2 Diabetes: A Retrospective Cross-Sectional Study**

Bing Liu^2#^, Jun Chen^3^, JingBo He^4^, Qing Xue^4^, Yue Liu^5^, Fei Gao^1^*, Hao Qi ^1*^

1. Department of Endocrinology, First Hospital of Shanxi Medical University, Taiyuan, Shanxi Province, China.

2. Department of Nuclear Medicine, First Hospital of Shanxi Medical University, Taiyuan, Shanxi Province, China.

3. Department of Endocrinology, Shanghai Pudong New Area People's Hospital, Shanghai 201299, China.

4. The Public Health College of Shanxi Medical University, Taiyuan, Shanxi Province, China.

5. The First Clinical Medical College of Shanxi Medical University, Taiyuan, Shanxi Province, China.

**Supplementary Table S1** Sex-stratified sensitivity analyses with additional adjustment for age (ordinal logistic regression)

## Table S1-A. Men

| Predictor | Model 1 + age OR (95% CI) | P | Model 2 + age OR (95% CI) | P | Model 3 + age OR (95% CI) | P |
| --- | --- | --- | --- | --- | --- | --- |
| Plaque (CP) vs No plaque (NCP) | 1.38 (0.90–2.13) | 0.141 | 1.30 (0.84–2.02) | 0.240 | 1.27 (0.82–1.97) | 0.292 |

**Footnote:** Ordinal logistic regression (proportional odds model),Model 1 + age: adjusted for diabetes duration, BMI, and age; Model 2 + age: additionally adjusted for TC, TG, HDL-C, LDL-C; Model 3 + age: additionally adjusted for FBG and HbA1c;Proportional odds assumption not violated (all P > 0.05) Odds ratios are presented for Plaque (CP) relative to No plaque (NCP) (reference).

## Table S1-B. Postmenopausal women

### Table S1-B1. Ordinal logistic regression（Model 1 + age）

| Predictor | Model 1 + age OR (95% CI) | P |
| --- | --- | --- |
| Plaque (CP) vs No plaque (NCP) | 0.89 (0.66–1.19) | 0.421 |

Footnote: Proportional odds assumption not violated (P = 0.455)

### Table S1-B2. Multinomial logistic regression(for models violating the proportional odds assumption)

| Outcome comparison | Model 2 + age OR (95% CI) | P | Model 3 + age OR (95% CI) | P |
| --- | --- | --- | --- | --- |
| ON vs NO | 0.79 (0.52–1.20) | 0.269 | 0.79 (0.52–1.20) | 0.269 |
| OP vs NO | 0.74 (0.48–1.14) | 0.176 | 0.74 (0.48–1.14) | 0.176 |

Footnote: Multinomial logistic regression was applied when the proportional odds assumption was violated (Model 2 + age and Model 3 + age);Reference category: NO; Adjusted for age, BMI, diabetes duration, lipid parameters, and glycemic indices

**Supplementary Table S2** Multinomial logistic regression analysis of the association between carotid plaque and osteoporosis status in women

| Outcome (ref=NO) | Model | OR (Plaque (CP) vs No plaque (NCP)) | 95% CI | P |
| --- | --- | --- | --- | --- |
| ON vs NO | Model 2 | 1.468 | 0.978–2.203 | 0.064 |
| OP vs NO | Model 2 | 1.761 | 1.171–2.646 | 0.007 |
| ON vs NO | Model 3 | 1.486 | 0.988–2.232 | 0.057 |
| OP vs NO | Model 3 | 1.808 | 1.199–2.725 | 0.005 |

**Footnote:** Multinomial logistic regression was performed as a sensitivity analysis due to violation of the proportional odds assumption in women. Normal bone mass (NO) was used as the reference outcome category. Odds ratios (ORs) are presented for carotid plaque (CP), with non-carotid plaque (NCP) as the reference exposure group.

**Supplementary Table S3.** Sex-stratified interaction analysis of serum 25(OH)D × calcium on BMD: model fit (reduced covariate set)

|  |  |  |  |  |  |  |  |  |
| --- | --- | --- | --- | --- | --- | --- | --- | --- |
| Outcome | Sex | Model 1 R² | Model 1Adj. R² | Model 2 R² | Model 2Adj. R² | ΔR² | F-change (df1,df2) | P for ΔR² |
| BMD | Men | 0.235 | 0.207 | 0.235 | 0.205 | 0.000 | 0.003 (1, 413) | 0.959 |
| BMD | Women | 0.218 | 0.203 | 0.219 | 0.202 | 0.000 | 0.205 (1, 775) | 0.651 |

**Footnote:** Model 1 adjusted for Age, BMI, SBP, DBP, Pulse, Duration, LDL, HDL, P, Mg, BGP, PTH, PINP, β-CTX, and HbA1c. Model 2 additionally included the standardized 25(OH)D× calcium interaction term (IntZ_25OHD_Ca).

**Supplementary Table S4.** Sex-stratified coefficient of the standardized vitamin D × calcium interaction term on BMD (reduced covariate set)

| Outcome | Sex | Interaction term | B (SE) | Beta | t | P | VIF |
| --- | --- | --- | --- | --- | --- | --- | --- |
| BMD | Men | IntZ_25OHD_Ca | 0.000 (0.004) | 0.002 | 0.051 | 0.959 | 1.127 |
| BMD | Women | IntZ_25OHD_Ca | 0.003 (0.006) | 0.015 | 0.452 | 0.651 | 1.034 |

**Footnote:** The interaction term was constructed using standardized (Z-score) 25(OH)D and calcium values. Multicollinearity was assessed using variance inflation factors (VIF).

**Supplementary Table S5.** Three-way interaction test (Sex × 25(OH)D × calcium) on BMD in the combined sample: hierarchical model fit (reduced covariate set)

| Outcome | Model | Block added | R² | Adj. R² | ΔR² | F-change (df1, df2) | P for ΔR² |
| --- | --- | --- | --- | --- | --- | --- | --- |
| BMD | 1 | Main effects (Sex, Z25(OH)D, ZCa  ) + covariates | 0.321 | 0.310 | 0.321 | 31.541 (18, 1203) | <0.001 |
| BMD | 2 | + 25(OH)D×  Ca(IntZ_25OHD_Ca) | 0.321 | 0.310 | 0.001 | 1.024 (1, 1202) | 0.312 |
| BMD | 3 | +Sex×25(OH)D× Ca (Sex_ IntZ) | 0.321 | 0.310 | 0.000 | 0.058 (1, 1201) | 0.809 |

**Footnote:** Sex was coded as 0=Men and 1=Women. Covariates included Age, BMI, SBP, DBP, Pulse, Duration, LDL, HDL, P, Mg, BGP, PTH, PINP, β-CTX, and HbA1c. 25(OH)D and calcium were standardized (Z-scores). IntZ_25OHD_Ca = Z25(OH)D × ZCa; Sex_IntZ = Sex × IntZ_25OHD_Ca.

**Supplementary Table S6.** Coefficients for the two-way and three-way interaction terms on BMD in the combined sample (reduced covariate set)

| Outcome | Term | B (SE) | Beta | t | P | VIF |
| --- | --- | --- | --- | --- | --- | --- |
| BMD | 25(OH)D× Ca (IntZ_25OHD_Ca) | -0.004 (0.004) | -0.042 | -1.039 | 0.299 | 2.910 |
| BMD | Sex×25(OH)D× Ca (Sex_ IntZ) | -0.003 (0.012) | -0.007 | -0.241 | 0.809 | 1.311 |

**Footnote:** Sex was coded as 0=Men and 1=Women. The three-way interaction term tests whether the 25(OH)D× Ca interaction differs by sex. VIF indicates variance inflation factor.

**Supplementary Table S7.** Vertebra-level sensitivity analyses for lumbar spine BMD to address potential DXA inflation from abdominal aortic calcification/degenerative changes (Mann–Whitney U test)

| Sex | Outcome | Mann–Whitney U | Z | P |
| --- | --- | --- | --- | --- |
| Men | Total lumbar spine BMD (L1–L4) | 15004.500 | -2.479 | 0.013 |
| Men | Mean BMD (L1–L2) | 15115.500 | -2.381 | 0.017 |
| Men | Mean BMD (L1–L3) | 15108.000 | -2.387 | 0.017 |
| Women | Total lumbar spine BMD (L1–L4) | 72843.000 | -1.076 | 0.282 |
| Women | Mean BMD (L1–L2) | 73042.500 | -1.084 | 0.279 |
| Women | Mean BMD (L1–L3) | 72921.500 | -1.122 | 0.262 |

**Footnote:** Plaque groups were compared using the Mann–Whitney U test. L1–L2 and L1–L3 mean BMD were calculated from vertebra-specific DXA measures to reduce potential inflation from abdominal aortic calcification/degenerative changes.
